# Clarithromycin-Induced Long QT Syndrome: A Case Report

**DOI:** 10.1155/2012/634652

**Published:** 2012-03-26

**Authors:** Mecnun Cetin, Munevver Yıldırımer, Serkan Özen, Sema Tanrıverdi, Senol Coskun

**Affiliations:** ^1^Division of Pediatric Cardiology, Department of Pediatrics, Faculty of Medicine, Celal Bayar University, 45010 Manisa, Turkey; ^2^Department of Pediatrics, Ağrı State Hospital, 04000 Ağrı, Turkey; ^3^Division of Neonatology, Department of Pediatrics, Faculty of Medicine, Celal Bayar University, 45010 Manisa, Turkey

## Abstract

Long QT syndrome develops for a number of reasons. The number of non-antiarrhythmic drugs reported to induce QT interval prolongation with or without torsade de pointes continues to increase. Clarithromycin is a macrolide antibiotic being increasingly used for the treatment of atypical pneumonia. In this paper, we describe a patient who developed long QT prolongation syndrome after receiving clarithromycin for the treatment of atypical pneumonia.

## 1. Introduction

Long QT syndrome (LQTS) is a repolarization disorder with idiopathic, iatrogenic, and congenital etiologies that may cause recurrent syncope attacks, life-threatening tachyarrhythmia, and sudden cardiac death [1, 2]. While hereditary form of LQTS is associated with mutations of the genes that are responsible for formation of ion channels, iatrogenic form is more often related to drugs and electrolyte imbalances. There are numerous drugs other than antiarrhythmic ones that may cause LQTS with or without torsade de pointes. Clarithromycin is a macrolide group antibiotic with extended spectrum of action. Although LQTS due to erythromycine has been reported frequently in the literature, LQTS caused by clarithromycin has been rarely reported [3, 4]. It is being used frequently for various indications in pediatric patients. Here we presented a case of LQTS developed during clarithromycine treatment which was prescribed for treating atypical pneumonia.

## 2. Case

**Figure 1:**
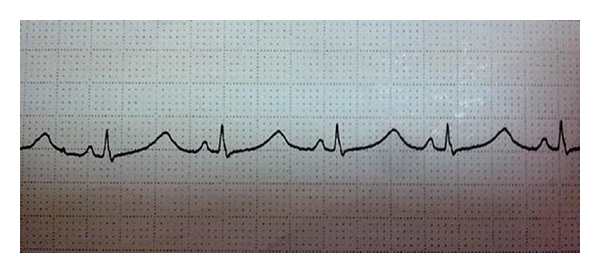
ECG of the patient (cQT 600 msec).

A 6-years-old male child who has been started on clarithromycine treatment with a diagnosis of atypical pneumonia 6 days ago was directed to our pediatric cardiology clinics as cardiac murmur was noticed during his physical examination. During taking his history we learned that he had a syncope attack of short duration 12 hours prior to admission to our clinic. He had no history of familial unexplained sudden death at younger ages or syncope attacks. In physical examination, his general condition was good, he was conscious, his cardiac rate and blood pressure were 94/min and 100/70 mmHg, respectively. There was a 2/6 systolic murmur located in mesocardiac area. Physical examination of other systems was normal. Laboratory results including CBC and biochemistry were normal. His ECG revealed sinüs rhythm, cardiac rate of 88/min, a normal axis, QRS duration of 60 msec, QT interval of 480 msec, and cQT interval of 600 msec which were long for his age, and no T-wave abnormality or dysrhythmia (Figure 1). Echocardiographic evaluation was normal. In his 24-hour Holter ECG monitorization, there were no rythm abnormalities other than long QT interval. As he had no family history, electrocardiographic evaluation of parents did not reveal long QT interval and audiometric evaluation of patient was normal, we excluded congenital LQTS. We thought clarithromycine that he has been using may be the etiologic factor and he stopped it. One week later his QT interval returned to normal (420 msec). Diagnosis became definite during the one year of follow-up period in which he had no syncope attacks and his ECGs remained normal.

## 3. Discussion

Long QT syndrome (LQTS) is a cardiac repolarization disorder with idiopathic, iatrogenic, and congenital etiologies that may cause recurrent syncope attacks, life-threatening tachyarrhythmia, and sudden cardiac death [1, 2]. Characteristic ECG findings are long cQT interval for age, T-wave abnormalities, and torsade de pointes-type ventricular tachyarrhythmias induced by exercise [3, 4]. Iatrogenic form is more often related to drugs and electrolyte imbalances. Although elongation of QT interval has been reported with antiarrhythmic drugs like quinidine, sotalol, amiodarone and procainamide, drugs other than antiarrhythmic ones including antihistaminics, cholinergic antagonists, antifungal drugs, antipsychotics, and various antibiotics (erythromycine, clarythromycine, cotrimoxazole) may cause torsade de pointes by elongating QT interval. Torsade de pointes cases due to drugs are thought to be associated with blockage of potassium channels [5]. Two cardinal findings of LQTS are syncope and abnormalities in ECG. Underlying cause of syncope attacks is cardiac arrythmias. Although we did not detect any dysrhythmias in ECG and 24-hour holter evaluation of our patient, we thought dysrhythmia as the probable underlying etiology for the syncope attack. Because clinical presentation of LQTS has been quite variable, Schwartz et al. [6] proposed some major and minor criteria which have been modified for children (Table 1). According to these criteria scores of less than 1, of 2-3, and of equal to and more than 4 are designated as low probability, medium probability, and high probability, respectively. Presentation findings of this syndrome are syncope attacks induced by exercise and fear. Routine ECG and evaluation of cQT may not be adequate sometimes. Along with that there may be T-wave abnormalities and notching on T wave. 24-hour Holter ECG monitorization may be useful for diagnosis [7]. As clarythromycine usage due to pneumonia and *Helicobacter* pylori eradication regimes has been increased, more cases of LQTS due to this antibiotic are being reported. Individuals with borderline QT interval value for their age and history of cardiac disease, antibiotics alternative to clarythromycine that does not cause LQTS should be chosen. But again normal QT intervals in an individual do not exclude the possibility for development of LQTS [8]. Clarythromycine is a frequently used drug in pediatric population for various indications. For that one must be cautious for its side effect. With this case we wanted to emphasize that when drugs causing elongation of QT interval are required to be used, prior electrocardiographic evaluation of the patients should be performed, and in individuals with long QT intervals these drugs should not be preferred if possible or they should be used with extreme caution with close ECG followup. In addition this case is remarkable as it shows that clarythromycine usage may result in serious life threatening side effects related to elongated cQT interval even in individuals with normal QT intervals.

## Figures and Tables

**Table 1:** Diagnostic criteria for LQTS.

ECG findings	
cQT	
≥480 msec	3 points
460–470 msec	2 points
450 msec (in males)	1 points
Torsade de pointes	2 points
T-wave alternans	1 points
Notched T wave in 3 derivation	1 points
Low heart rate for age	0.5 points

Clinical history	
Syncope	
With stress	2 points
Without stress	1 points
Congenital deafness	0.5 points

Family history	
Siblings with LQTS	1 points
History of sudden death before 30 years of age	0.5 points
